# Characterization of size-fractionated carbonaceous particles in the small to nano-size range in Batam city, Indonesia

**DOI:** 10.1016/j.heliyon.2023.e15936

**Published:** 2023-04-29

**Authors:** Muhammad Amin, Gita Prajati, Gita Pati Humairoh, Rahmi Mulia Putri, Worradorn Phairuang, Mitsuhiko Hata, Masami Furuuchi

**Affiliations:** a Faculty of Geosciences and Civil Engineering, Institute of Science and Engineering, Kanazawa University, Kanazawa, Ishikawa, 920-1192, Japan; b Faculty of Engineering, Maritim University of Raja Ali Haji, Tanjung Pinang, Kepulauan Riau, 29115, Indonesia; c Environmental Engineering Department, Universitas Universal, Batam, Kepulauan Riau, 29456, Indonesia; d Graduate School of Natural Science and Technology, Kanazawa University, Kanazawa, Ishikawa, 920-1192, Japan; e Faculty of Environmental Management, Prince of Songkla University, Hat Yai, Songkhla, 90110, Thailand

**Keywords:** Ultrafine particles, COVID-19 pandemic, Organic carbon, Elemental carbon, Local emission, Long-range transportation, Indonesia

## Abstract

A cascade impactor type sampler equipped with an inertial filter was used to collect size-segregated particles down to ultrafine particles (UFPs or PM_0.1_) on Batam Island in Sumatra, Indonesia, bordered by Singapore and Malaysia during a wet and the COVID-19 pandemic season in 2021. Carbonaceous species, including organic carbon (OC) and elemental carbon (EC), were analyzed by a thermal/optical carbon analyzer to determine the carbon species and their indices. The average UFP was 3.1 ± 0.9 μg/m^3^, which was 2–4 times lower than in other cities in Sumatra during the same season in the normal condition. The PMs mass concentration was largely affected by local emissions but long-range transportation of particles from Singapore and Malaysia was also not negligible. The air mass arrived at the sampling site passed the ocean, which introduced out clean air with a low level of PMs. The backward trajectory of the air mass and the largest fraction of OC2 and OC3 in all sizes was identified as being transported from the 2 above countries. OC is the dominant fraction in TC and the ratio of carbonaceous components indicated that origin of all particle sizes was predominantly vehicle emissions. UFPs were dominantly emitted from vehicles exhaust emission, while coarser particles (>10 μm) were influenced by the non-exhaust emissions, such as tire wear. Other particles (0.5–1.0; 1.0–2.5; and 2.5–10 μm) were slightly affected by biomass burning. The effective carbon ratio (ECR) and inhalation dose (ID) related EC indicated that finer particles or UFPs and PM_0.5-1_ contributed more to human health and global warming.

## Highlights

• UFPs were first evaluated in Batam, a small island nearby Singapore and Malaysia.
• PMs in Batam Island were influenced by local and transboundary emission.
• Vehicle emission was the primary source of UFPs in Batam.
• OC2 and OC3 were the dominant fractions of TC.

## Graphical abstract

**Figure:**
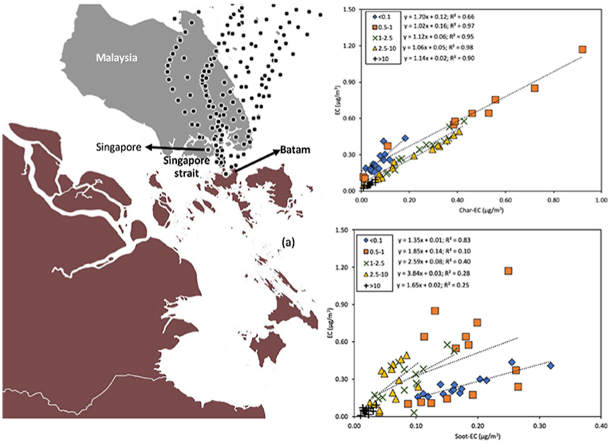

## 1 Introduction

It is well known that carbonaceous components are major contributors to particulate matter (PMs). Liang et al. (2016) reported that such components account for 10–70% of the fine particles worldwide, and Putri et al. (2021) reported that carbon components make up for 25–50% of ultrafine particles (UFPs) in urban areas [1,2]. Atmospheric particulate carbon is a complex mixture of carbon-containing compounds that are typically divided into two major fractions: elemental carbon (EC) and organic carbon (OC) [[3], [4], [5]]. OC accounts for the organic fraction of carbonaceous material. Based on the sources of these materials, they can be divided into primary or secondary organic carbon (POC or SOC). POC is produced as the result of the incomplete combustion of organic materials such as biomass burning and fossil fuel. It can also be produced by the degradation of carbon-containing products such as vegetation, while SOC is commonly formed by aqueous-phase and photochemical reactions through gas-to-particle conversion [6,7]. On the other hand, EC only emitted from the primary sources during the incomplete combustion of biomass fires and fossil fuels [[8], [9], [10]]. Particles that contain OC is involved in cooling the atmosphere because it reflects and scatters solar radiation [11]. Recent publications have even revealed that some OC i.e., brown carbon (BrC) could absorb the light at 300–400 nm and could inhibit the cooling action [[12], [13], [14], [15]]. On the other hand, EC has the capacity to absorb sunlight and generate a warming effect [16].

Since they have affected various activities of human life, numerous studies concerning carbonaceous components have been reported world-wide for several types of environments in attempts to obtain more information regarding their sources and their effects [[17], [18], [19]]. However, most of these studies have focused on East Asia, Europe, and America. Only limited numbers of investigations related to carbonaceous components that are generated in the Southeast Asia (SEA) region, particularly in Indonesia, have appeared [2,[20], [21], [22], [23]]. For example, information on the size distribution of carbonaceous components, mainly down to UFP or PM_0.1_ remains very limited. The smaller particles are of greater concern than larger particles due to their greater impact on human health. These particles can penetrate deeper into the respiratory system. For example, larger particles (>10 μm) are retained in the oropharyngeal region and the larynx due to impaction. When particles of 5–10 μm are inhaled, they are largely deposited in the airways while the particle with sizes of 1–2.5 μm can be deposited in the bronchioles. Particle smaller than 1 μm remains longer and easily penetrate into the alveoli. UFPs mainly enter the human body through the lungs and are then translocated to all other essential organs [[24], [25], [26]].

Batam city is a highly urbanized and industrialized city in Indonesia. Because of its location bordering Singapore and Malaysia, in 1994, the Sijori Growth Triangle was established to enhance economic activity between Singapore, Malaysia (Johor Bahru), and Indonesia (Riau archipelago province, particularly Batam city) [27]. As a result, Batam city has the second highest number of tourist arrivals after Bali Island, which has led to increased economic activities. The increasing economic activities in Batam are in line with the growing use of fossil fuels for cooking, industrial use, and transportation, leading to decreased air quality. However, stations for monitoring air quality have not been developed in Batam as in Singapore and Malaysia, which has made air quality assessment in this region a challenge.

In this study, we attempted to fill this gap by carrying out extensive studies with the primary purposes of: (a) understanding the distribution of PMs including UFP and the carbonaceous components in Batam Island (b) to identify the sources of these carbonaceous components in the PMs by measuring OC/EC ratios, developing OC/EC vs EC correlations, soot-EC/TC ratios along with data on air mass trajectory provided by NASA (c) to evaluate the effect of the COVID-19 pandemic at the PMs level in Batam Island (d) to estimate the effects of carbonaceous aerosols on global warming and exposure risk to human health.

## 2 Methodology

### 2.1 Site description

**Fig. 1:**
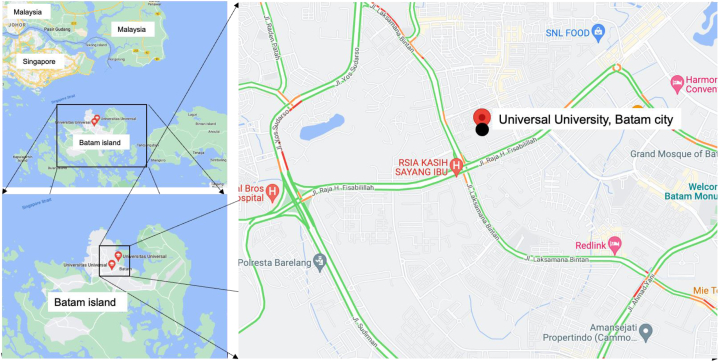
Sampling sites located in Batam Island nearby Singapore and Malaysia.

The sampling site, displayed in Fig. 1, is located at the Universal University (1°07′45.9″N 104°02′01.2″E), in Batam city. Batam is an industrially important and a tourism city located on Batam island, Riau archipelago province, Indonesia. Geographically, Batam island is one of the most strategic areas in SEA since it is located on the international shipping route. The land area of Batam is around 1038 km^2^ while the ocean area is about 2791 km^2^. Similar to the other cities in Sumatera Island, Batam has a tropical climate. The minimum and maximum ranges for temperature are 20.7–23.9 °C and 26.8–29.1 °C, respectively. Atmospheric pressure in Batam recorded at 1010.6 mb (minimum) and at 1013.5 mb (maximum) and the humidity level ranges from 75% to 86% [27]. The sampling of size distribution of PMs including PM_0.1_ was conducted from March to April in 2021.

The wind direction particularly in March and April is largely from the northeast and southwest with an average wind velocity of 2.5 and 1.76 m/s, respectively [27]. The level of precipitation in both months was 209.4 and 131 mm^3^. Based on census data collected in 2020, the total population of municipal Batam was 1,196,396, with a population growth rate per year was 2.32% [27].

### 2.2 Sampling methods

The PMs were collected by using a cascade-type air sampler, i.e., an Ambient Nano sampler (ANS) which consists of 4-impactor stages to collect different sizes of particles using a quartz fiber filter (QFF) (diameter = 55 mm; Pallflex, 2500 QAT- UP), (>10, 10–2.5, 2.5–1, 1–0.5 μm) from the upper to the lower parts, respectively [28]. The next stage consists of an inertial filter (IF) (SUS304, fiber diameter = 9.8 μm) [29] to classify the particles 0.5–1 μm, and finally, the last stage is used to collect the particles smaller than 100 nm or PM_0.1_ by using the same types and size of QFF [30]. To remove possible contaminants, the QFF filters were pre-baked before being used at a temperature 350 °C following the Ministry of Environment of Japan (MOEJ) [31] procedures. If the temperature is too high (>500 °C), the surface of the fiber filters will be more active and allow the OC to be more easily absorbed by the filters, especially after the cooling down process [32].

Furthermore, after the baking process, the filter was then stored in the weighing chamber for 48 h which operated at both a stable temperature and humidity at 21.5 ± 1.5 °C and 35 ± 5%, respectively, before and after sampling. 75 QFFs and 14 IFs were used from March 13th to April 16th, 2021. Each filter was covered with aluminum foil then stored in zip-lockck plastic bag. A travel blank (n = 3) was also stored in the same plastic bag with the filter to evaluate the possible contamination during transport to/from the sampling sites.

### 2.3 Carbon analyses

Carbonaceous components (OC and EC) were analyzed by means of a thermal/optical carbon analyzer (Model 5L, Sunset Instruments Inc., USA) followed by the Interagency Monitoring of Protected Visual Environments-Thermal/Optical Reflectance (IMPROVE-TOR) method. The QFFs were punched into 1.5 cm^2^ sections The temperature of the analyzer was gradually increased to 120, 250, 450, 550 °C in a non-oxidizing oven to evaluate the OC1, OC2 OC3, and OC4, respectively. An oxidizing atmosphere of oxygen (O_2_, 2%) and helium (He, 98%) was then added to the oven and the temperature was then increased to 550, 700, and 800 °C for the classification of EC1, EC2, and EC3, respectively. Pyrolysis organic carbon (PyOC) was determined as the carbon that was combusted after the O_2_ was added to the experimental atmosphere. The total OC is defined as the sum up of all OC fractions + PyOC while the total EC as EC1+EC2+EC3 minus by PyOC. Char-EC and soot-EC also determined in this study. Char-EC is defined as EC1-PyOC while soot EC is defined as EC2 + EC3. Total carbon (TC = OC + EC) was also evaluated along with the PMs to understand the fraction of total carbon in each particle size.

For the quality assurance and quality control (QA/QC) of carbon analyses, the carbon analyzer was first calibrated by using reference standards (sucrose; C_12_H_22_O_11_(196-00015, Sucrose, Wako Pure Chemical Industries, Ltd., Japan)) and a blank filter was used to determine the minimum detection limit (MDLs) which is 0.03 μg/m^3^ and 0.00 for OC and EC, respectively, which suggests that its value was small enough as a blank filter in this study. IFs and QFFs with the spot samples obtained by a multi-nozzle cascade impactor originally could not analyzed since it was not applicable for use in thermal/optical methods. However, regarding the QFFs spot samples, we analyzed these by adjusting the punched area at the same location as accurately as possible and to confirm this, we calculated the repeatability of the analyses of spot samples. We concluded that this was reasonable as a CV of less than 3.2% for the OC and 7.9 for the EC was obtained.

### 2.4 Backward air mass trajectory

The 72-h backward trajectory arrived at the sampling site in Batam island was calculated by using HYbrid Single-Particle Lagrangian Integrated Trajectory Model version-4 (HYSPLIT4) developed by NOAA′s Air Resources Laboratory [33,34]. The height of the arrival level to the sampling site was set at 500 m from the above ground level as a compromise between the limitations of the model and the data observed at the ground surface [35].

## 3 Results and discussion

### 3.1 Mass concentration of size-fractionated PMs

**Fig. 2:**
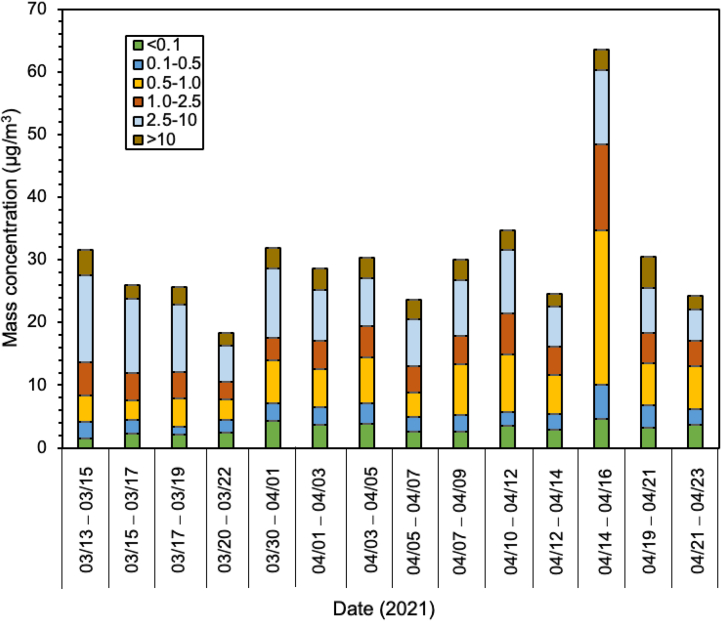
Mass concentration of size-fractionated of PMs in Batam Island, Indonesia.

**Fig. 3:**
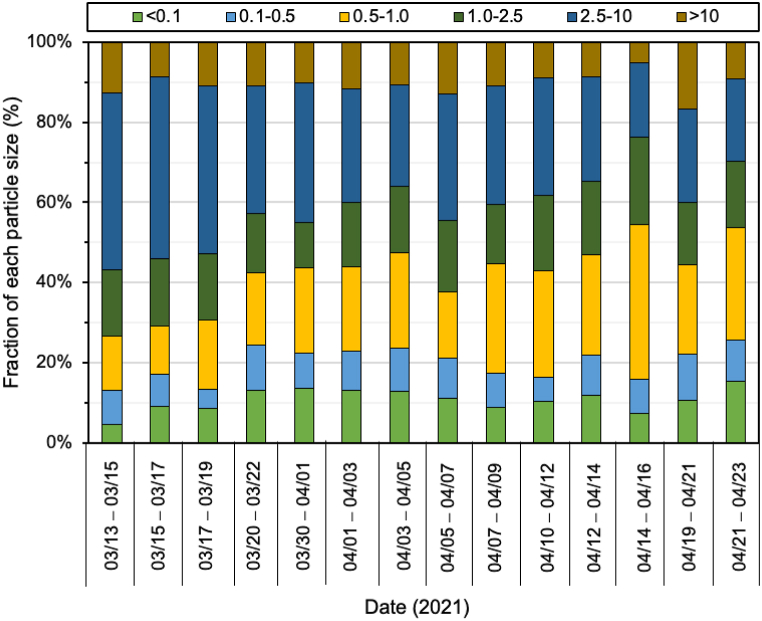
Mass fraction of PMs in Batam Island, Indonesia.

The daily concentration of each size of PM and its average are displayed in Fig. 2. The exact value for PM and their ratios are listed in Table 1. In Fig. 3, the particle sizes fraction in the total suspended particulate (TSP) is shown. The average of the PM_0.1_ at 3.1 ± 0.9 μg/m^3^ in Batam Island was considerably lower than in other cities on Sumatera Island, Indonesia. Compared to the data reported at the same season i.e., rainy season, Indonesia reported by Amin et al. [22] and Putri et al. [2], the PM_0.1_ level in Batam Island was 2–4 times lower than that in Padang, Medan, and Jambi, city (Table 2).

**Table 1:** Mass concentration of size-fractionated of PMs and their ratio in Batam Island.

Size	03/13–03/15	03/15–03/17	03/17–03/19	03/20–03/22	03/30–04/01	04/01–04/03	04/03–04/05	04/05–04/07	04/07–04/09	04/10–04/12	04/12–04/14	04/14–04/16	04/19–04/21	04/21–04/23	Ave ± stdev
Size-segregate of PM (μg/m^3^)															
<0.1	1.42	2.32	2.17	2.39	4.32	3.71	3.87	2.63	2.66	3.56	2.93	4.67	3.24	3.72	3.11 ± 0.9
0.1–0.5	2.68	2.11	1.26	2.08	2.78	2.84	3.31	2.35	2.57	2.11	2.44	5.33	3.54	2.48	2.71 ± 0.9
0.5–1.0	4.29	3.10	4.45	3.33	6.82	6.00	7.23	3.91	8.16	9.22	6.23	24.64	6.78	6.86	7.21 ± 5.1
1.0–2.5	5.27	4.36	4.23	2.70	3.61	4.57	5.04	4.21	4.46	6.51	4.51	13.82	4.80	3.98	5.15 ± 2.5
2.5–10	13.90	11.82	10.76	5.88	11.15	8.10	7.68	7.45	8.90	10.20	6.41	11.80	7.09	5.03	9.01 ± 2.5
>10	4.03	2.23	2.83	2.00	3.24	3.36	3.24	3.06	3.29	3.12	2.13	3.20	5.12	2.21	3.07 ± 0.8
PMs (μg/m^3^)															
PM_1_	8.38	7.53	7.88	7.80	13.91	12.54	14.41	8.89	13.39	14.90	11.59	34.65	13.55	13.06	13.03 ± 6.5
PM_2.5_	13.65	11.89	12.10	10.50	17.52	17.12	19.45	13.10	17.84	21.41	16.10	48.47	18.35	17.04	18.18 ± 8.9
PM_10_	27.55	23.71	22.86	16.38	28.67	25.22	27.13	20.55	26.74	31.61	22.51	60.27	25.44	22.07	27.19 ± 9.9
TSP	31.58	25.94	25.69	18.37	31.91	28.58	30.37	23.61	30.03	34.73	24.64	63.47	30.56	24.28	30.27 ± 10.1
PMs ratio (−)															
PM_0.1_/PM_1_	0.17	0.31	0.28	0.31	0.31	0.30	0.27	0.30	0.20	0.24	0.25	0.13	0.24	0.28	0.26 ± 0.1
PM_0.1_/PM_2.5_	0.10	0.20	0.18	0.23	0.25	0.22	0.20	0.20	0.15	0.17	0.18	0.10	0.18	0.22	0.18 ± 0.0
PM_0.1_/PM_10_	0.05	0.10	0.10	0.15	0.15	0.15	0.14	0.13	0.10	0.11	0.13	0.08	0.13	0.17	0.12 ± 0.0
PM_0.1_/TSP	0.04	0.09	0.08	0.13	0.14	0.13	0.13	0.11	0.09	0.10	0.12	0.07	0.11	0.15	0.11 ± 0.0
PM_1_/PM_2.5_	0.61	0.63	0.65	0.74	0.79	0.73	0.74	0.68	0.75	0.70	0.72	0.71	0.74	0.77	0.71 ± 0.1
PM_1_/PM_10_	0.30	0.32	0.34	0.48	0.49	0.50	0.53	0.43	0.50	0.47	0.52	0.57	0.53	0.59	0.47 ± 0.1
PM_1_/TSP	0.27	0.29	0.31	0.42	0.44	0.44	0.47	0.38	0.45	0.43	0.47	0.55	0.44	0.54	0.42 ± 0.1
PM_2.5_/PM_10_	0.50	0.50	0.53	0.64	0.61	0.68	0.72	0.64	0.67	0.68	0.72	0.80	0.72	0.77	0.65 ± 0.1
PM_2.5_/TSP	0.43	0.46	0.47	0.57	0.55	0.60	0.64	0.55	0.59	0.62	0.65	0.76	0.60	0.70	0.59 ± 0.1
PM_10_/TSP	0.87	0.91	0.89	0.89	0.90	0.88	0.89	0.87	0.89	0.91	0.91	0.95	0.83	0.91	0.89 ± 0.0

**Table 2:** Mass fraction of PM_0.1_ in TSP in several cities in Indonesia and Asian.

Location	Description	PM_0.1_/TSP (%)	Reference
Batam, Indonesia	Urban, (rainy season)	10.68	This study
Medan, Indonesia	Rural, (volcano area)	18.68	[2]
	Urban, (roadside)	14.36	
	Urban, (school closed to roadside)	12.73	
	Urban, (industrial area)	5.68	
Padang, Indonesia	Rural, (rainy season)	14.90	[23]
	Rural, dry season	15.53	
Jambi, Indonesia	Suburban (rainy season)	16.93	
	Suburban (dry season)	13.66	
Pekanbaru, Indonesia	Urban, (rainy season)	17.80	
	Urban, (dry season)	16.35	
Padang, Indonesia	Residential area nearby cement industry and roadside	18.71	[36]
Suzu, Japan	Urban	8.35	[37]
Toyama, Japan	Urban	14.00	
Kanazawa, Japan	Mixed	8.38	
Pathumtani	Suburban (rainy season)	13.68	[38]
Thailand	Suburban (dry season)	15.11	

In the dry season where smoke-haze commonly occurs, especially in the peatland area along the east coast of Sumatera Island and open burning of agricultural residues resulted in higher level of PMs, and the PM_0.1_ levels were substantially higher around 4–6 times than PM_0.1_ values in Batam city [21,22]. The present study was comparable with the PM_0.1_ data in developed countries or in several cities in Japan (Table 2) [[36], [37], [38]]. Particles with sizes of 2.5–10 μm were the highest fraction among all sizes and accounted for 30.8 ± 8% (18.6–45.6%) followed by particles with sizes of 0.5–1 μm (22.3 ± 6.7%). The same trend was found in all cities in southeast Asia (SEA). Fine particles, <2.5 μm accounted for around 59% of TSP. Considering that Batam Island is larger than Padang city and Muaro Jambi regency, taking into account the population and number of vehicle users, one of the reasons for the lower fraction of fine particles compared to other cities might be due to the covid-19 pandemic [[39], [40], [41]]. During the sampling period, Batam island was in lockdown and the government permitted only limited outdoor activities. Since the citizens were forced to stay at home, vehicle use was probably decreased, and this would have affected the concentrations of all sizes of PMs. The number of vessels operating from/to Batam, and Singapore was also decreased due to lockdown regulations in both regions (see. Fig. S1). This might be one of the reasons for the lowest PM concentration in this region. Moreover, as shown in Fig. S2, most of the air mass that arrived at the sampling site Batam Island came from Malaysia and Singapore and then passed over the ocean that generally cleaned this carried air.

### 3.2 The sources of carbonaceous components of PMs

**Fig. 4:**
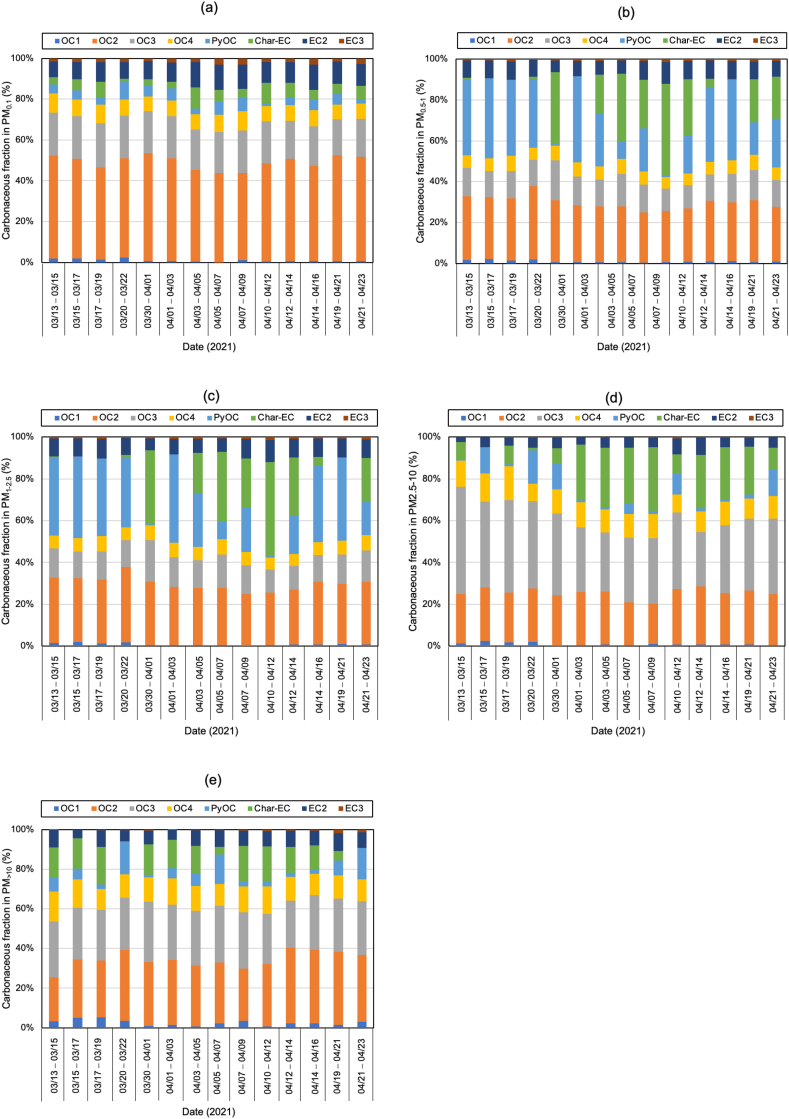
Carbonaceous fraction of PMs in Batam Island, Indonesia (a) PM_0.1_ (b) PM_0.5-1_ (c) PM_1-2.5_ (d) PM_2.5-10_ (e) PM_>10_.

Fig. 4 displays the carbonaceous fraction of OC1, OC2, OC3, OC4, PyOC, Char-EC, EC2, and EC3 in Batam island. Each carbon component has a unique source and can be used as the indicator of a specific emission source. Based on previous studies, OC1 is emitted from biomass burning, OC2 is aging particles derived from secondary products during long-range transportation on the ocean surface, OC3 is commonly related to exhaust from gasoline powered engines and also from biomass burning, while OC4 mainly originates from the road-dust during the movement of vehicles [[42], [43], [44], [45], [46], [47], [48]]. It is well known that PyOC is derived from the decomposition of OC during thermal analyses to form EC, which is commonly generated from cooking and biomass burning. EC1 is predominantly emitted from coal, biomass burning, and from gasoline engines, while EC2 and EC3 are emitted from diesel and gasoline engines [47,48].

**Fig. 5:**
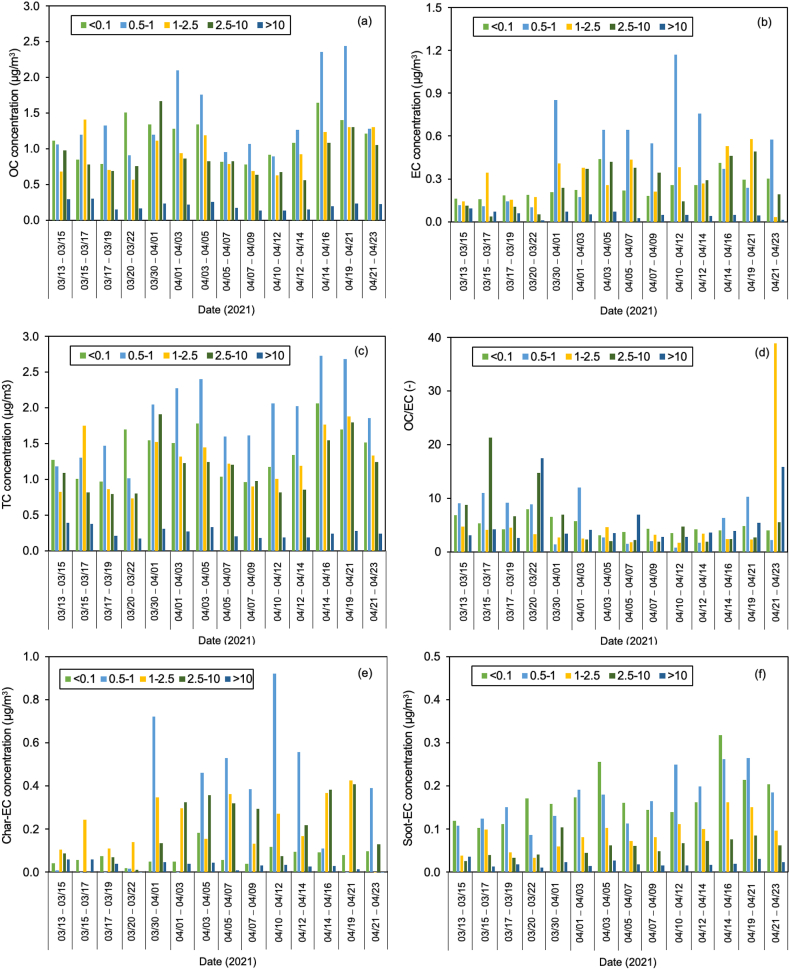

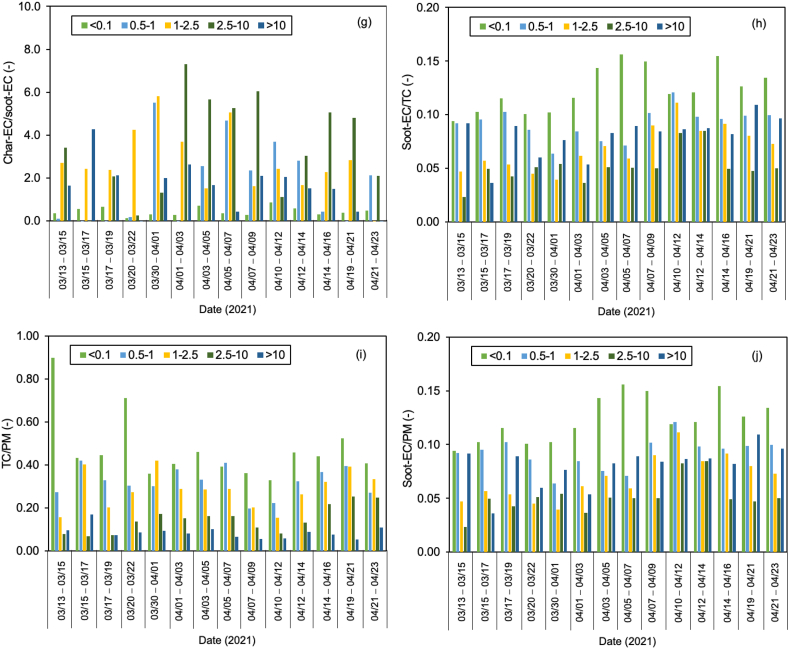
Carbonaceous concentration and its ratio in Batam city, Indonesia (a) OC, (b) EC, (c) TC, (d) OC/EC (e) char-EC (f) soot-EC (g) char-EC/soot-EC (h) soot-EC/TC (i) TC/PM (j) soot-EC/PM.

**Fig. 6:**
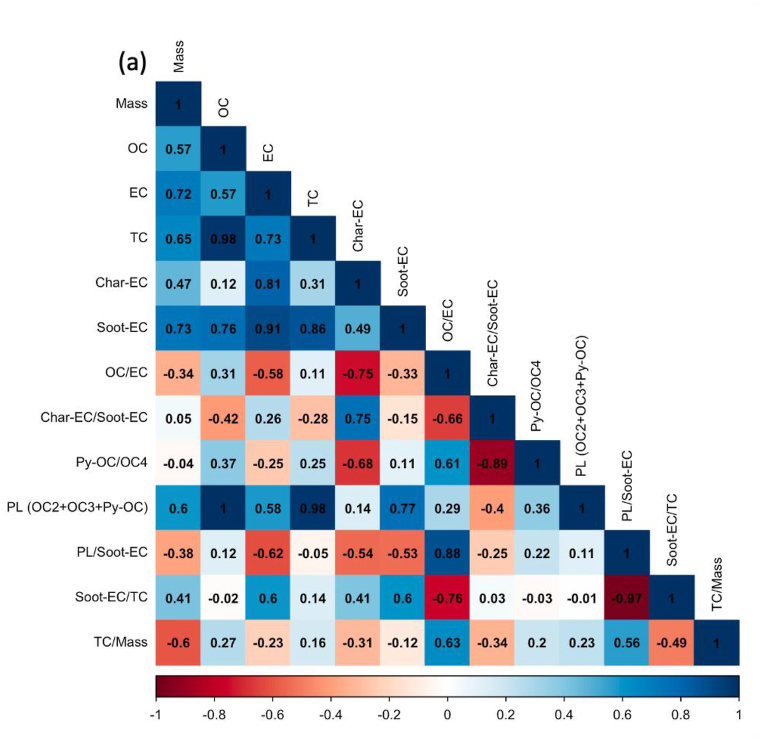

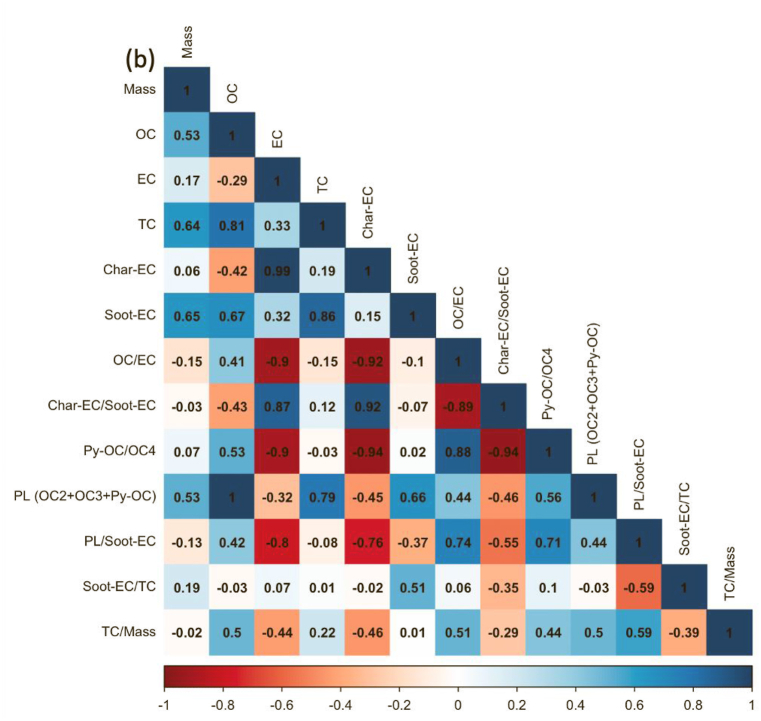

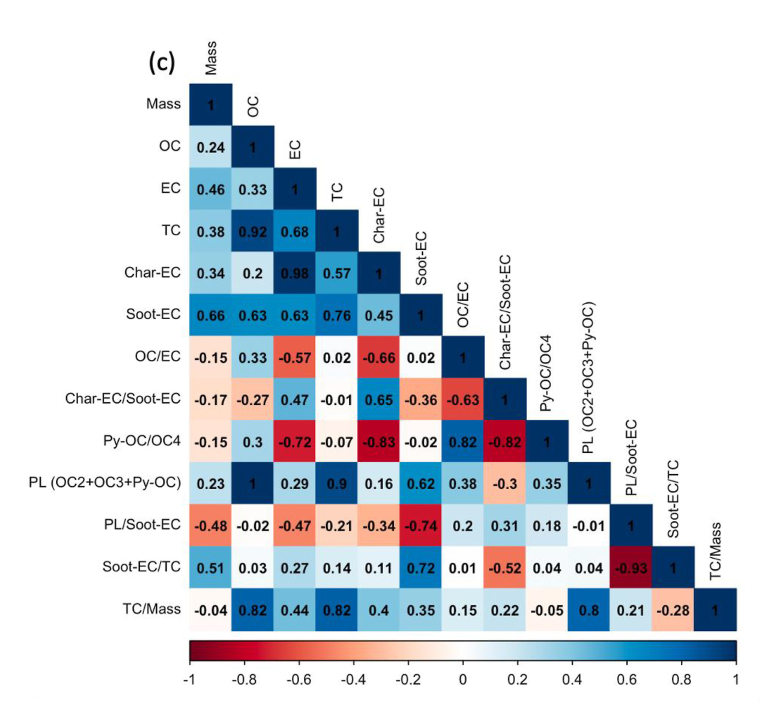

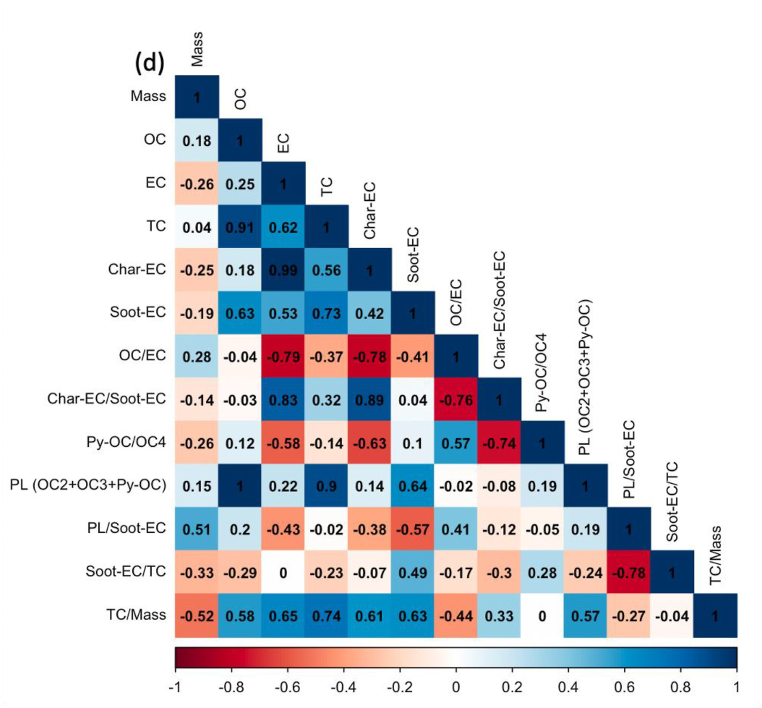

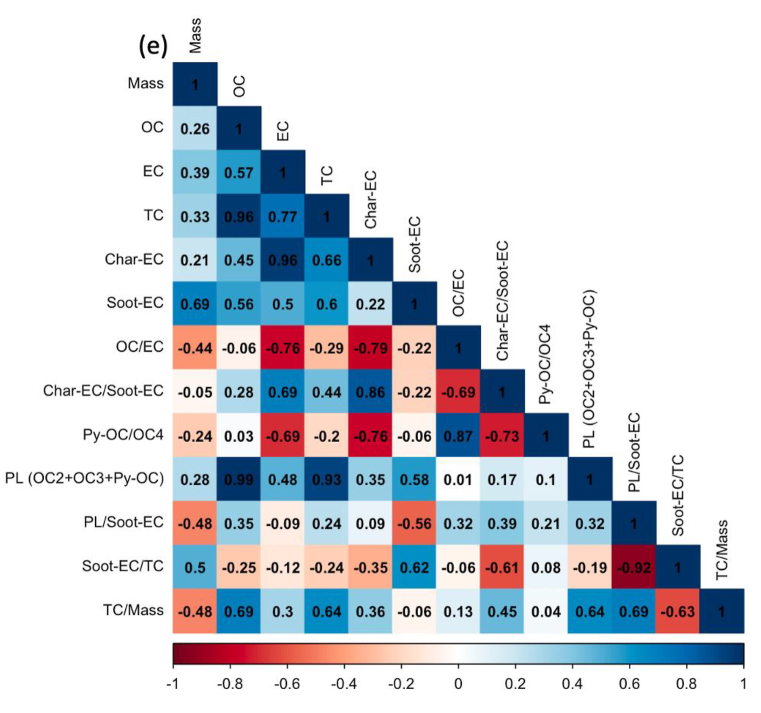
Carbon correlation of PMs in Batam island, Indonesia (a) PM_0.1_ (b) PM_0.5-1_ (c) PM_1-2.5_ (d) PM_2.5-10_ (e) PM_>10_ Sources: Medan: Putri et al., 2021 Padang, Jambi, Pekanbaru: Amin et al., 2021.

Regardless of the day of sampling, OC2 and OC3 are the highest fractions of carbon, accounting for more than half of the total carbon, especially ultrafine particles. The values for OC2 and OC3 were about 48.2 ± 3.2 and 20.1 ± 1.1%, respectively, suggesting that they are secondary products of long-range transportation on the ocean and vehicle emissions, especially from gasoline exhaust (see Fig. S1). As stated in section 2.1. Batam city is located on Batam island nearby Singapore and is surrounded by ocean. Fig. 5 shows the concentration of carbonaceous components and their ratio, and Fig. 6(a–e) displays the Pearson correlation between parameters in all particle sizes or indoor for PM_0.1_, PM_0.5–1_, PM_1–2.5_, PM_2.5–10_, and PM_>10_. OC and char-EC were higher in the case of 0.5–1, 1–2.5, and 2.5–10 μm particles and might be associated with biomass burning [2,21,22]. These values are in line with the insignificant correlation between OC and EC in these particle sizes, suggesting that OC and EC originated from different sources, particularly PM_0.5–1._ The highest soot-EC was found in ultrafine particles associated with emissions from vehicle combustion. It was decreased along an increase in particles sizes due to exhaust from vehicles that emitted smaller size particles [49,50]. The higher soot-EC/TC in coarser particles (>2.5 μm) might be due to road-dust and tire wear during the vehicle movement (breaking) around the sampling site since the OC4/TC ratio was higher in the coarser particle as shown in Table 3 [51]. Carbonaceous components accounted for more than half of the ultrafine particles in entire sampling period. This is consistent with a previous study concerning the SEA. The Char-EC/soot-EC ratio in fine and coarse particles that were higher than unity indicated increased primary emission from biomass and coal combustion, whereas the lowest ratio was found to be PM_0.1_ (less than unity) suggesting a substantial contribution by automobile and motorcycle emissions [34,36]. It also can be seen from Fig. 6 that shows a better correlation of soot-EC vs EC rather than char-EC vs EC in PM_0.1_. On the other hand, the char-EC vs. EC correlation in other sizes of particles was better than soot-EC vs. EC.

**Table 3:** The average of carbonaceous component and their ratio in Batam Island, Indonesia.

Size	OC (μg/m^3^)	EC (μg/m^3^)	TC (μg/m^3^)	OC/EC (−)	soot-EC (μg/m^3^)	soot-EC/TC (−)	OC4/TC (−)	soot-EC/PM (−)	TC/PM (−)
<0.1	1.15 ± 0.29	0.25 ± 0.09	1.40 ± 0.034	4.89 ± 1.42	0.17 ± 0.06	0.12 ± 0.02	0.08 ± 0.1	0.12 ± 0.02	0.47 ± 0.15
	0.78–1.65	0.16–0.44	0.96–2.06	3.07–7.96	0.10–0.32	0.09–0.16	0.07–0.09	0.09–0.16	0.33–0.90
0.5–1	1.41 ± 0.53	0.46 ± 0.33	1.87 ± 0.54	5.64 ± 4.24	0.17 ± 0.06	0.09 ± 0.01	0.07 ± 0.01	0.09 ± 0.01	0.32 ± 0.07
	0.89–2.44	0.10–1.17	1.01–2.73	0.76–12.01	0.09–0.27	0.06–0.12	0.06–0.07	0.06–0.12	0.20–0.42
1–2.5	0.96 ± 0.29	0.31 ± 0.16	1.27 ± 0.37	5.71 ± 9.60	0.09 ± 0.04	0.07 ± 0.02	0.10 ± 0.01	0.07 ± 0.02	0.28 ± 0.09
	0.56–1.41	0.03–0.58	0.74–1.88	1.64–38.88	0.03–0.16	0.04–0.11	0.09–0.12	0.04–0.11	0.15–0.42
2.5–10	0.91 ± 0.30	0.26 ± 0.16	1.17 ± 0.37	5.99 ± 5.71	0.06 ± 0.02	0.05 ± 0.02	0.11 ± 0.02	0.05 ± 0.02	0.15 ± 0.06
	0.56–1.41	0.04–0.49	0.79–1.91	1.84–21.27	0.03–0.10	0.02–0.08	0.08–0.16	0.02–0.08	0.07–0.25
>10	0.20 ± 0.06	0.05 ± 0.02	0.25 ± 0.07	5.69 ± 4.78	0.02 ± 0.01	0.08 ± 0.02	0.12 ± 0.01	0.08 ± 0.02	0.09 ± 0.03
	0.13–0.30	0.01–0.09	0.17–0.39	2.59–17.42	0.01–0.04	0.04–0.11	0.11–0.15	0.04–0.11	0.05–0.17

**Fig. 7:**
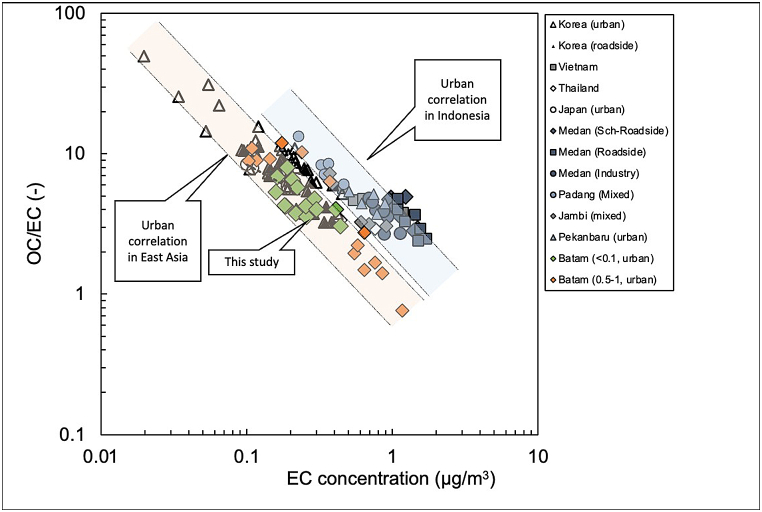
OC/EC vs EC correlation in urban area of different cities in East Asia and Indonesia compared to present study.

The OC/EC vs. EC correlation could be used to determine whether ultrafine particles more affected by local emissions from vehicles or whether they were influenced by biomass burning as reported by Amin et al. [21,22] and Putri et al. [2]. We plotted our data with previous studies in SEA (Indonesia, Thailand, Vietnam) and East Asia (Japan and South Korea) in Fig. 6. All the data were taken from urban sites. As can be seen from Fig. 7 the PM_0.1_ data in Batam city, which was closer to East Asian than Indonesian urban sites can be attributed to the characteristics of the city. Batam city is an industrial and tourism city and there is no agricultural area in this region as reported by statistical agency of Batam city, 2021 [27]. Hence, PM_0.1_ in Batam city is predominantly emitted from vehicle combustion rather than from fresh biomass burning.

### 3.3 Potential effect of carbonaceous components

#### 3.3.1 Estimation of potential exposure risk to EC

As explained previously, carbonaceous components have an adverse effect both on human health and the environment. Numerous previous studies have attempted to estimate the exposure of PMs and its chemical composition to the human body. Most of these studies involved heavy metals. However, in this study we used the EC data to calculate the total of EC concentration that is inhaled by a human, which be more illustrative of the potential effects of carbonaceous aerosols to the human body. EC was selected rather than OC due the fact that its sources only originated from primary sources and its more persistent in the atmosphere than OC. To calculate the exposure risk, we used an inhalation dose of EC based on the following equation:

$$Inhalation\;Dose\;\left(D\right)=Cp\;x\;{IR}_{\left(\Delta t\right)}x\;dt$$

Where *D* is the total inhalation dose (μg) of EC, *Cp* is the concentration of EC (μg/m^3^), *IR* is the inhalation rate of human (average IR for males and females with ages of 16–61 years is 0.0111 m^3^/min while for the children with age 6–15, the IR for male and female is 0.0094 m^3^/min) and *dt* is the exposure time of a person spending outside (8 h was used as reported previously as the minimum time spent by a person outdoors) [52].

In this study, the estimated inhalation dose for PMs was higher in PM_0.5–1_ and accounted for 2.45 ± 1.77 μg and 2.08 ± 1.50 μg for adults and children respectively. In ultrafine particles or PM_0.1,_ the inhalation doses of EC for adult and children were 1.32 ± 0.46 μg and 1.12 ± 0.39 μg. Detail information regarding this is listed in Table S1. Since, ultrafine particles can penetrate into the deepest part of the human respiratory system, they would be expected to be more harmful, and the sources of emitted ultrafine particles as vehicles emission, should be limited and further regulated to prevent the adverse effects of carbon aerosols on the human body. As reported by Zhang et al. [53] and by Barraza-Villarreal et al. [54], one of the factors for the high death and mortality rates was exposure to high levels of EC. Both short and long-term exposure to EC could play a role in the development of various diseases particularly acute myocardial infarction and even causing death.

#### 3.3.2 Estimation of the potential effect to the climate

Carbonaceous aerosols not only affect human health but also the climate since it could change the global temperature by absorbing and scattering light. The scientific community recently reached a consensus concerning carbonaceous aerosol particles in that they play an important role in climate change. In the introduction, we stated that carbon aerosols composed of OC could scatter light and EC or that black carbon (BC) could absorb light. EC and BC are defined by the method of analysis being employed. However, the OC/EC ratio in some cases does not provide accurate information on their impact and their sources since OC could be generated from both primary and secondary sources (POC and SOC). Both are emitted via different mechanisms and different interactions with solar radiation. As reported by Safai et al. [55], the effective carbon ratio (ECR) is more susceptible for the evaluation of the potential effect of carbonaceous aerosols on climate change. ECR could be calculated from the ratio of SOC/(POC + EC). Both POC and EC are able to absorb the light which is commonly emitted from the combustion of fossil fuels, agricultural burning, and domestic cooking, whereas SOC can scatter light that is mainly emitted from the oxidation of volatile organic compounds. The averages for SOC in the TC for all particle sizes were 27.5 ± 15.6; 57.7 ± 28.6; 36.9 ± 23.6; 38.8 ± 31.8; 30.8 ± 25.4% respectively for <0.1; 0.5–1; 1–2.5; 2.5–10; and >10 μm.

The lower ECR value suggests higher levels of POC and EC, a higher contribution of carbonaceous aerosol to global warming [56]. Table S1 lists the ECR values for five different sizes of PMs. The highest ERC was found in particle sizes of 0.5–1 and 1–2.5 μm at 2.77 ± 2.41 and 1.55 ± 3.64, indicating that these sizes of particles have a greater effect on cooling the atmosphere. On the other hand, the ECR value for ultrafine particles was the lowest. This is reasonable since it is predominantly emitted from vehicles with some contribution from biomass burning. A high level of EC and POC in PM_0.1_ could have a more harmful effect on human health and also result in enhancing the rate of global warming.

There are some uncertainties and limitations in the discussion regarding the effect of carbon components on the climate. For example, recently, some organic carbon, i.e., brown carbon (BrC), could contribute to the warming atmosphere due to its characteristic of absorbing light. Furthermore, more studies are important to understand the distribution of size-segregated PMs and their carbonaceous components in Batam Island and Indonesia during the different seasons, especially during post-pandemic COVID-19. The simulation using WRF-Chem could give more understanding regarding the impact of carbonaceous components on the climate. The combination between ground and data as emission datasets in CHIMERE could give more knowledge about health risk estimation regarding elemental carbon in Indonesia.

## 4 Conclusion

This study provides the first inventory of atmospheric aerosol bound carbonaceous components in Batam island. Compared to other cities during the same season, the UFP concentration in this study was much lower. While this study reports on the concentration of PMs in a type of Industrial city bordered with Singapore and Malaysia, it should be noted that the sampling process was conducted during the COVID-19 pandemic during which most of the citizens were working and students were studying from home and the air mass arriving to Batam Island was mostly transported thorough the ocean with a low loading of PMs, so the concentration of PMs and the related carbonaceous component in this study can be attributed to localized emissions in the vicinity of the Island. However, the long-range transport from Singapore and Malaysia could not be ignored. OC2 and OC3 is the dominant fraction in TC regardless the size of the PMs. The carbonaceous ratio indicates that particles with sizes of 0.5–1; 1–2.5; and 2.5–10, appear to be due to biomass burning while UFP was dominantly influenced by traffic emissions. Furthermore, non-vehicle emissions such as tire wear from braking contributed to the coarser particles or >10 μm, as indicated by the higher ratio of soot-EC/TC. Regarding the effect of carbon components, particularly EC, this study found that smaller particles, i.e., <0.1 and 0.5–1 μm, were inhaled at the highest doses by citizens and both of these sizes also contribute to global warming, and that <0.1 μm (UFP) contributes more to temperature increases, while 0.5–1 μm appears to contribute to the coolness of the atmosphere.

## Author contribution statement

Muhammad Amin: Conceived and designed the experiments; analyzed and interpreted the data; contributed reagents, materials, analysis data; wrote the paper.

Gita Prajati: Performed the experiment, Contributed reagents, materials, analysis data.

Gita Pati Humairoh: Performed the experiment, Contributed reagents, materials, analysis data.

Rahmi Mulia Putri: Conceived and designed the experiments, contributed reagents, materials, analysis data.

Worradorn Phairuang: Analyzed and interpreted the data, wrote the paper.

Mitsuhiko Hata: Conceived and designed the experiments; analyzed and interpreted the data.

Masami Furuuchi: Conceived and designed the experiments; analyzed and interpreted the data, wrote the paper.

All authors listed have significantly contributed to the development and the writing of this article.

## Funding statement

This work was financially supported by 10.13039/501100001691JSPS KAKENHI 21H03618, and 10.13039/100008608Sumitomo Foundation, Japan. Moreover, this work was partially supported by JICA-JST SATREPS (Grant No. JPMJSA2102).

## Data availability statement

No data was used for the research described in the article.

## Declaration of interest's statement

The authors declare no competing interests.

## Additional information

Supplementary content related to this article has been published online at [URL].

## Declaration of competing interest

The authors declare that they have no known competing financial interests or personal relationships that could have appeared to influence the work reported in this paper.
