# Supplementary material for: Characterization of size-fractionated carbonaceous particles in the small to nano-size range in Batam city, Indonesia
Source: Heliyon. 2023 Apr 29;9(5):e15936. doi: 10.1016/j.heliyon.2023.e15936 (PMC10192538; doi:10.1016/j.heliyon.2023.e15936)
Supplement: Multimedia component 1 [file mmc1.docx]

**List of supplementary materials**

**Characterization of size-fractionated carbonaceous particles in the small to**

**nano-size range in Batam city, Indonesia**

Muhammad Amin^a,b^, Gita Prajati^c^, Gita Pati Humairoh^c^, Rahmi Mulia Putri^d^,

Worradorn Phairuang^a^, Mitsuhiko Hata^a^, and Masami Furuuchi^a,e*^

^a^Faculty of Geosciences and Civil Engineering, Institute of Science and Engineering, Kanazawa University, Kanazawa, Ishikawa, 920-1192, Japan

^b^Faculty of Engineering, Maritim University of Raja Ali Haji, Tanjung Pinang,Kepulauan Riau 29115, Indonesia

^c^Environmental Engineering Department, Universitas Universal, Batam, Kepulauan Riau, 29456, Indonesia

^d^Graduate School of Natural Science and Technology, Kanazawa University, Kanazawa, Ishikawa, 920-1192, Japan

^e^Faculty of Environmental Management, Prince of Songkla University, Hat Yai, Songkhla, 90110, Thailand

**Corresponding author:** mfuruch@staff.kanazawa-u.ac.jp

**Fig. S1.** Number of vessels arrived at Singapore port (a) all types of vessels from 2017–2020 (b) passenger vessel from Batam to Singapore in 2020 and 2021

**Fig. S2.** Backward air mass trajectory arrived at sampling site in Batam city

**Table S1.** Effective carbon ratio (ECR) and inhalation dose based on elemental carbon in Batam city, Indonesia

(a)

(b)

**Fig. S1.** Number of vessels arrived at Singapore port (a) all types of vessels from 2017–2020 (b) passenger vessel from Batam to Singapore in 2020 and 2021

**
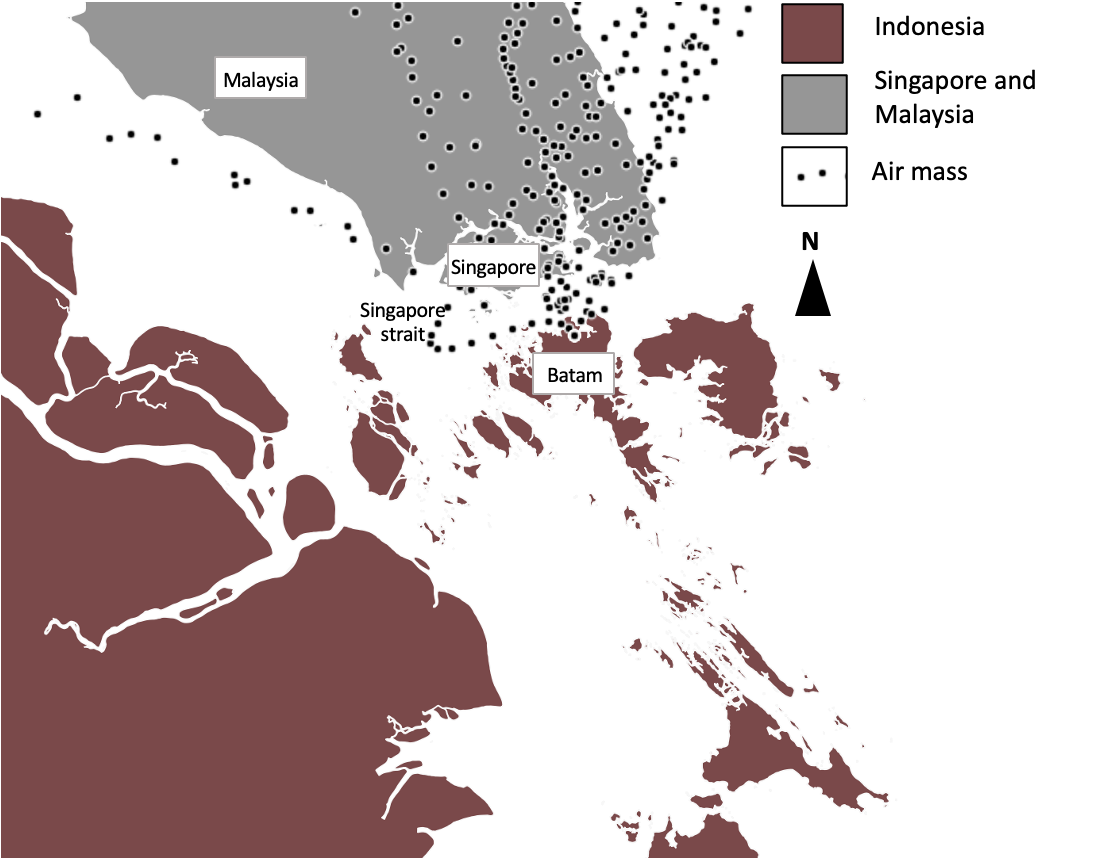
**

**Fig. S2.** Backward air mass trajectory arrived at sampling site in Batam city.

Description: Mostly air mass come to the sampling site was originated from Singapore and Malaysia

**Table S1.** Effective carbon ratio (ECR) and inhalation dose based on elemental carbon in Batam city, Indonesia

| <0.1 μm | POC | SOC | ERC = SOC/POC+EC | Inhalation Dose (D) (Adult, 16–61 years old)(ug) | Inhalation Dose (D) (Children, 6–15 years old)(ug) |
| --- | --- | --- | --- | --- | --- |
| 03/13 ⎼ 03/15 | 0.50 | 0.61 | 0.93 | 0.86 | 0.73 |
| 03/15 ⎼ 03/17 | 0.49 | 0.36 | 0.56 | 0.85 | 0.72 |
| 03/17 ⎼ 03/19 | 0.57 | 0.22 | 0.29 | 0.99 | 0.83 |
| 03/20 ⎼ 03/22 | 0.58 | 0.93 | 1.20 | 1.01 | 0.85 |
| 03/30 ⎼ 04/01 | 0.63 | 0.71 | 0.85 | 1.10 | 0.93 |
| 04/01 ⎼ 04/03 | 0.68 | 0.60 | 0.66 | 1.19 | 1.01 |
| 04/03 ⎼ 04/05 | 1.34 | 0.00 | 0.00 | 2.33 | 1.97 |
| 04/05 ⎼ 04/07 | 0.67 | 0.15 | 0.17 | 1.16 | 0.98 |
| 04/07 ⎼ 04/09 | 0.56 | 0.22 | 0.30 | 0.97 | 0.82 |
| 04/10 ⎼ 04/12 | 0.79 | 0.12 | 0.12 | 1.37 | 1.16 |
| 04/12 ⎼ 04/14 | 0.78 | 0.30 | 0.29 | 1.36 | 1.15 |
| 04/14 ⎼ 04/16 | 1.26 | 0.39 | 0.23 | 2.19 | 1.85 |
| 04/19 ⎼ 04/21 | 0.90 | 0.51 | 0.42 | 1.56 | 1.32 |
| 04/21 ⎼ 04/23 | 0.93 | 0.29 | 0.23 | 1.61 | 1.36 |
| average | 0.76  ±0.27 | 0.39  ±0.26 | 0.45  ±0.35 | 1.32  ±0.46 | 1.12  ±0.39 |
| 0.5–1 μm | POC | SOC | ERC = SOC/POC+EC | Inhalation Dose (D) (Adult, 16–61 years old)(ug) | Inhalation Dose (D) (Children, 6–15 years old)(ug) |
| 03/13 ⎼ 03/15 | 0.09 | 0.97 | 4.70 | 0.63 | 0.53 |
| 03/15 ⎼ 03/17 | 0.08 | 1.11 | 5.81 | 0.58 | 0.49 |
| 03/17 ⎼ 03/19 | 0.11 | 1.21 | 4.79 | 0.77 | 0.65 |
| 03/20 ⎼ 03/22 | 0.08 | 0.83 | 4.59 | 0.55 | 0.46 |
| 03/30 ⎼ 04/01 | 0.65 | 0.55 | 0.36 | 4.54 | 3.84 |
| 04/01 ⎼ 04/03 | 0.13 | 1.97 | 6.38 | 0.93 | 0.79 |
| 04/03 ⎼ 04/05 | 0.49 | 1.27 | 1.12 | 3.42 | 2.90 |
| 04/05 ⎼ 04/07 | 0.49 | 0.46 | 0.41 | 3.43 | 2.90 |
| 04/07 ⎼ 04/09 | 0.42 | 0.65 | 0.67 | 2.92 | 2.47 |
| 04/10 ⎼ 04/12 | 0.89 | 0.00 | 0.00 | 6.23 | 5.28 |
| 04/12 ⎼ 04/14 | 0.58 | 0.69 | 0.52 | 4.03 | 3.41 |
| 04/14 ⎼ 04/16 | 0.28 | 2.07 | 3.16 | 1.98 | 1.68 |
| 04/19 ⎼ 04/21 | 0.18 | 2.26 | 5.38 | 1.27 | 1.08 |
| 04/21 ⎼ 04/23 | 0.44 | 0.84 | 0.83 | 3.06 | 2.59 |
| average | 0.35  ±0.25 | 1.06  ±0.65 | 2.77  ±0.41 | 2.45  ±1.77 | 2.08  ±1.50 |
| 1–2.5 μm | POC | SOC | ERC = SOC/POC+EC | Inhalation Dose (D) (Adult, 16–61 years old)(μg) | Inhalation Dose (D) (Children, 6–15 years old)(μg) |
| 03/13 ⎼ 03/15 | 0.24 | 0.45 | 1.18 | 0.77 | 0.65 |
| 03/15 ⎼ 03/17 | 0.56 | 0.85 | 0.95 | 1.82 | 1.54 |
| 03/17 ⎼ 03/19 | 0.25 | 0.45 | 1.10 | 0.83 | 0.70 |
| 03/20 ⎼ 03/22 | 0.28 | 0.28 | 0.61 | 0.92 | 0.78 |
| 03/30 ⎼ 04/01 | 0.67 | 0.45 | 0.42 | 2.17 | 1.84 |
| 04/01 ⎼ 04/03 | 0.62 | 0.32 | 0.32 | 2.02 | 1.71 |
| 04/03 ⎼ 04/05 | 0.42 | 0.76 | 1.12 | 1.38 | 1.16 |
| 04/05 ⎼ 04/07 | 0.71 | 0.07 | 0.06 | 2.32 | 1.97 |
| 04/07 ⎼ 04/09 | 0.35 | 0.34 | 0.61 | 1.13 | 0.96 |
| 04/10 ⎼ 04/12 | 0.63 | 0.00 | 0.00 | 2.04 | 1.73 |
| 04/12 ⎼ 04/14 | 0.44 | 0.48 | 0.68 | 1.43 | 1.21 |
| 04/14 ⎼ 04/16 | 0.86 | 0.37 | 0.27 | 2.81 | 2.38 |
| 04/19 ⎼ 04/21 | 0.94 | 0.36 | 0.23 | 3.08 | 2.61 |
| 04/21 ⎼ 04/23 | 0.05 | 1.25 | 14.13 | 0.18 | 0.15 |
| average | 0.50  ±0.25 | 0.46  ±0.32 | 1.55  ±3.64 | 1.64  ±0.83 | 1.38  ±0.70 |
| 2.5–10 μm | POC | SOC | ERC = SOC/POC+EC | Inhalation Dose (D) (Adult, 16–61 years old)(ug) | Inhalation Dose (D) (Children, 6–15 years old)(ug) |
| 03/13 ⎼ 03/15 | 0.20 | 0.77 | 2.45 | 0.59 | 0.50 |
| 03/15 ⎼ 03/17 | 0.07 | 0.71 | 6.84 | 0.19 | 0.16 |
| 03/17 ⎼ 03/19 | 0.19 | 0.50 | 1.70 | 0.55 | 0.47 |
| 03/20 ⎼ 03/22 | 0.09 | 0.66 | 4.55 | 0.27 | 0.23 |
| 03/30 ⎼ 04/01 | 0.44 | 1.23 | 1.81 | 1.27 | 1.08 |
| 04/01 ⎼ 04/03 | 0.68 | 0.18 | 0.17 | 1.97 | 1.67 |
| 04/03 ⎼ 04/05 | 0.77 | 0.05 | 0.04 | 2.23 | 1.89 |
| 04/05 ⎼ 04/07 | 0.70 | 0.13 | 0.12 | 2.02 | 1.71 |
| 04/07 ⎼ 04/09 | 0.63 | 0.00 | 0.00 | 1.83 | 1.55 |
| 04/10 ⎼ 04/12 | 0.26 | 0.41 | 1.02 | 0.76 | 0.64 |
| 04/12 ⎼ 04/14 | 0.54 | 0.02 | 0.03 | 1.55 | 1.32 |
| 04/14 ⎼ 04/16 | 0.85 | 0.24 | 0.18 | 2.45 | 2.08 |
| 04/19 ⎼ 04/21 | 0.90 | 0.40 | 0.29 | 2.62 | 2.22 |
| 04/21 ⎼ 04/23 | 0.35 | 0.70 | 1.28 | 1.02 | 0.86 |
| average | 0.48  ±0.29 | 0.43  ±0.36 | 1.46  ±2.01 | 1.38  ±0.83 | 1.17  ±0.70 |
| >10 μm | POC | SOC | ERC = SOC/POC+EC | Inhalation Dose (D) (Adult, 16–61 years old)(ug) | Inhalation Dose (D) (Children, 6–15 years old)(ug) |
| 03/13 ⎼ 03/15 | 0.24 | 0.05 | 0.15 | 0.50 | 0.42 |
| 03/15 ⎼ 03/17 | 0.19 | 0.12 | 0.46 | 0.38 | 0.32 |
| 03/17 ⎼ 03/19 | 0.15 | 0.00 | 0.00 | 0.31 | 0.26 |
| 03/20 ⎼ 03/22 | 0.02 | 0.14 | 4.13 | 0.05 | 0.04 |
| 03/30 ⎼ 04/01 | 0.18 | 0.06 | 0.22 | 0.37 | 0.31 |
| 04/01 ⎼ 04/03 | 0.14 | 0.08 | 0.43 | 0.28 | 0.24 |
| 04/03 ⎼ 04/05 | 0.19 | 0.07 | 0.26 | 0.38 | 0.33 |
| 04/05 ⎼ 04/07 | 0.07 | 0.11 | 1.20 | 0.13 | 0.11 |
| 04/07 ⎼ 04/09 | 0.12 | 0.01 | 0.07 | 0.25 | 0.21 |
| 04/10 ⎼ 04/12 | 0.13 | 0.01 | 0.06 | 0.26 | 0.22 |
| 04/12 ⎼ 04/14 | 0.11 | 0.04 | 0.27 | 0.22 | 0.19 |
| 04/14 ⎼ 04/16 | 0.13 | 0.06 | 0.36 | 0.26 | 0.22 |
| 04/19 ⎼ 04/21 | 0.11 | 0.12 | 0.78 | 0.23 | 0.20 |
| 04/21 ⎼ 04/23 | 0.04 | 0.19 | 3.68 | 0.08 | 0.06 |
| average | 0.13  ±0.06 | 0.08  ±0.05 | 0.86  ±1.33 | 0.27  ±0.12 | 0.22  ±0.10 |
